# Clinical characteristics and local recurrence risk in patients with multiple actinic keratoses: a retrospective clinical data analysis

**DOI:** 10.3389/fonc.2026.1770341

**Published:** 2026-02-11

**Authors:** Zhaogui Liu, Yaping Tang, Ping He, Jian Long, Zhang Chen, Miao Wan, Jianjian Zhu

**Affiliations:** 1 Department of Dermatology, Changde Hospital, Xiangya School of Medicine, Central South University (The First People’s Hospital of Changde City), Changde, Hunan, China; 2 Department of Dermatology, The Tenth Affiliated Hospital of Southern Medical University (Dongguan City People's Hospital), Dongguan, China

**Keywords:** actinic keratosis, Cox regression, nomogram, prediction model, recurrence

## Abstract

**Objective:**

To evaluate the risk of local recurrence in patients with multiple actinic keratoses (AKs), to analyze the influence of host-, lesion-, and treatment-related factors, and to develop and validate a Cox regression model for predicting recurrence risk.

**Methods:**

This retrospective study enrolled 148 patients with multiple AKs during January 2019-February 2022. Baseline characteristics, treatment modalities, and follow-up outcomes were collected. The Kaplan-Meier method was used to estimate cumulative recurrence rates. Cox regression analyses were performed to identify independent risk factors. The interaction effect between lesion count and treatment response was further assessed. A multivariate Cox predictive model was constructed, and its performance was validated using the 24-month calibration curve, Harrell’s C-index, and Brier score. A nomogram was developed for individualized risk prediction.

**Results:**

The cumulative recurrence rates at 12, 24, and 36 months were 22.3%, 35.6%, and 44.7%, respectively. Multivariate analysis identified higher lesion count (11-20: HR = 2.39; > 20: HR = 2.96) and incomplete treatment response (HR = 2.43) as independent risk factors. Immunosuppression and regular sunscreen use were not significant. Although visual analysis suggested elevated risk with more lesions and incomplete response, their interaction term was not statistically significant. The model demonstrated moderate discrimination (C-index = 0.630) and good calibration (Brier score = 0.176). The nomogram enabled individualized risk estimation.

**Conclusion:**

Lesion burden and incomplete treatment response significantly predict recurrence in multiple AKs patients. The developed Cox model and nomogram offer a clinically useful tool for identifying high-risk individuals and optimizing management strategies.

## 1 Introduction

Actinic keratosis (AK) is a chronic cutaneous photodamage condition induced by long-term ultraviolet (UV) radiation exposure, representing an early stage in the development of squamous cell carcinoma (SCC) or a precancerous lesion. Its histological hallmark is atypical hyperplasia of epidermal keratinocytes, clinically presenting as scaly or crusted patches (1). Epidemiological studies indicate that the prevalence of AK increases significantly with age, particularly in individuals with fair skin, chronic sun exposure, and those who are immunosuppressed (2). With an aging population and cumulative photodamage, AK has become one of the most common precancerous skin lesions worldwide.

Clinically, patients with multiple AKs often present with numerous lesions, and the affected skin areas frequently exhibit a state termed “field cancerization”. This concept posits that within sun-exposed skin, even apparently normal surface areas may harbor underlying genetic mutations and cellular damage, predisposing the field to frequent local recurrences and new lesion development (3). Research indicates that multiple AKs not only signify a higher cumulative burden of photodamage but are also closely associated with an increased risk of local recurrence and progression to SCC (4).

Current treatment modalities for AK are diverse, including cryotherapy, photodynamic therapy (PDT), topical 5-fluorouracil (5-FU), imiquimod, and various combination or sequential regimens (5). Although these methods can effectively clear lesions in the short term, recurrence remains a significant challenge in clinical management. Previous studies suggest that recurrence is influenced not only by the treatment modality but also by multiple factors, including host factors [e.g., immunosuppression, skin phototype, history of non-melanoma skin cancer (NMSC)], lesion characteristics (e.g., number, location, thickness/morphology), and sun protection behaviors (6). However, systematic studies assessing the risk of recurrence in patients with multiple AKs remain limited, particularly regarding long-term follow-up data stratified by lesion number and location characteristics. Therefore, this study, based on a retrospective analysis of clinical data from patients with multiple AKs, aims to systematically evaluate the relationship between host-, lesion-, and treatment-related characteristics and the risk of local recurrence.

## 2 Materials and methods

### 2.1 Study population

This single-center retrospective cohort study included 148 AK patients who visited the Department of Dermatology at our hospital and received standard treatment between January 2019 and February 2022. All cases were clinically and dermoscopically diagnosed by at least two senior dermatologists, with pathological confirmation when necessary. The study was approved by the Ethics Committee of Changde Hospital, Xiangya School of Medicine, Central South University (The First People’s Hospital of Changde City) and adhered to the principles of the *Declaration of Helsinki*. Due to the retrospective nature of the study and the absence of privacy risks, the Ethics Committee waived the requirement for informed consent. All data were de-identified and anonymized prior to analysis.

Inclusion criteria (1): Aged ≥ 18 years (2); Clinically confirmed multiple AKs (≥ 5 lesions per patient) (3); Received systematic and standardized initial treatment at our center (including local laser therapy, PDT, oral retinoids, imiquimod, or combination/sequential regimens) (4); Completed at least 12 months of clinical follow-up post-treatment with trackable recurrence status (5); Possessed complete clinical data providing the main study variables.

Exclusion criteria (1): Lacking key exposure or outcome information (2); Diagnosed with *in situ* or invasive SCC requiring surgical excision at initial presentation (3); Comorbid severe systemic diseases or conditions affecting follow-up (e.g., active malignancy, severe immunodeficiency) (4); Lost to follow-up or incomplete data preventing determination of recurrence time.

The primary outcome was the time to local recurrence, analyzed using the Cox proportional hazards regression model. The required number of events (E) was calculated using the formula: $E=\frac{{\left(Z_{1-\alpha /2}+Z_{1-\beta }\right)}^{2}}{{\left[ln(HR)\right]}^{2}\times p\times \left(1-p\right)}$, where α is the two-sided significance level (set at 0.05, Z_1−_α/_2_ = 1.96), β is the type II error rate (set at 0.20, power = 80%), HR is the expected hazard ratio, and *p* is the proportion in the exposed group. Based on previous research on AK recurrence (7), HRs for various factors typically range from 1.8-2.0. Assuming HR = 2.0 and *p* = 0.5, approximately 65 events were required. Considering the adjustment for multiple confounders in the multivariate Cox model and Peduzzi’s rule (requiring ≥ 10 events per predictor variable), and planning to include 6–7 primary variables, the total number of events should ideally be ≥ 70. Given reported 1–3 year local recurrence rates of 25%-35% for multiple AKs, a sample size (N) of approximately 233 patients was theoretically needed to achieve 80% power. However, the final cohort comprised 148 patients. With an expected recurrence rate of 30%, this yielded approximately 44 events, providing moderate statistical power (≈ 70%) for analyzing primary risk factors, while subgroup and interaction analyses should be considered exploratory.

### 2.2 Data collection and variable definitions

Clinical data for all enrolled patients were sourced from the hospital’s electronic medical record system and outpatient follow-up records. Data extraction followed a standardized protocol, with dual independent entry and cross-verification to ensure accuracy. Data extraction covered the period from the initial visit to the last follow-up. Variables collected included:

#### 2.2.1 Host factors

Age, sex, skin phototype (Fitzpatrick classification, grouped into light and dark types), immunosuppression status (organ transplant, long-term immunosuppressive medication), and history of NMSC.

#### 2.2.2 Lesion factors

Number of lesions (categorized as 5–10, 11–20, and >20), primary lesion location (head/scalp, forearm/dorsal hand, trunk or lower limbs), and lesion morphology assessed by clinical examination and dermoscopy. Lesions were classified as thin or non-thin according to the Olsen clinical grading system: thin lesions corresponded to Olsen grade I actinic keratoses, characterized by slightly palpable erythematous macules or patches with minimal hyperkeratosis, whereas non-thin lesions included Olsen grade II–III lesions, presenting as moderately to markedly hyperkeratotic, clearly palpable lesions. When grading discrepancies occurred, consensus was reached by two senior dermatologists.

#### 2.2.3 Treatment-related factors

Initial treatment modality (local laser therapy, PDT, oral retinoids, topical imiquimod, or combination/sequential therapy), treatment course completion (yes/no), and treatment response (complete or partial). Treatment response (8) was defined as: Complete clearance: disappearance of all clinically visible AKs in the treated area; Partial clearance: reduction in lesion count by ≥75%.

#### 2.2.4 Protective factor

Regular sunscreen use (yes/no).

#### 2.2.5 Recurrence definition and determination

Local recurrence was defined as the reappearance of a clinically (with dermoscopic correlation) or pathologically confirmed AK lesion within a 2 cm radius of the completely healed treatment area, assessed at least 1 month post-treatment completion. The recurrence time was calculated from the end of initial treatment to the date of first confirmed recurrence. For patients without recurrence, the last follow-up date or study cutoff date was used as the censoring time. Cumulative recurrence rates at 12, 24, and 36 months were recorded for survival analysis.

### 2.3 Data quality control

All data were independently verified and entered by two researchers, with discrepancies resolved by a senior dermatologist. The extent and pattern of missing data were assessed before analysis. Variables with more than 5% missing data included regular sunscreen use (6.8%) and lesion morphology (5.4%), while all other variables had missingness below 5%.

Missing values were assumed to be missing at random and were handled using multiple imputation by chained equations (MICE). A total of five imputations were generated, incorporating all variables included in the Cox regression models and the outcome indicator. Estimates from the imputed datasets were pooled according to Rubin’s rules for subsequent analyses.

### 2.4 Statistical analysis

Data were analyzed using R software (version 4.3.0). A two-sided *P*-value< 0.05 was considered statistically significant. Continuous variables were tested for normality. Normally distributed data were presented as mean ± standard deviation ( $\bar{\text{x}}$ ± s) and compared using the *t*-test, while non-normally distributed data were presented as median (interquartile range) and compared using the Wilcoxon rank-sum test. Categorical variables were presented as frequencies and percentages, and compared using the *χ²* test or Fisher’s exact test. Local recurrence was the endpoint event. The Kaplan-Meier method was used to plot recurrence-free survival (RFS) curves and calculate cumulative recurrence rates at 12, 24, and 36 months, with inter-group comparisons using the log-rank test. Cox proportional hazards regression models were used to analyze factors influencing local recurrence. Univariate analysis included all clinical variables, calculating HRs and 95% confidence intervals (CIs). Variables with clinical importance or P< 0.10 in univariate analysis were considered for inclusion in the multivariable model, with careful attention to model parsimony. Subgroup and interaction analyses were performed to assess result consistency across populations. Considering the relatively limited number of recurrence events, the number of covariates included in the multivariable Cox model was restricted based on clinical relevance and events-per-variable considerations to minimize the risk of overfitting. Interaction analyses were performed for exploratory purposes only and were not used for model selection. The proportional hazards assumption was verified using Schoenfeld residual tests. All tests were two-sided.

## 3 Results

### 3.1 Baseline characteristics

A total of 148 patients with multiple AKs met the inclusion criteria. The cohort comprised 81 males (54.73%) and 67 females (45.27%), with a mean age of 69.43 ± 7.58 years. During follow-up, 54 patients (36.49%) experienced local recurrence. Patients were divided into recurrence (n = 54) and non-recurrence (n = 94) groups. No significant differences were observed between groups regarding age, sex, skin phototype, immunosuppression status, or history of NMSC (all *P* > 0.05). Lesion number was closely associated with recurrence: the recurrence group had a higher lesion count (15.76 ± 8.57 vs. 13.03 ± 7.81, *P* = 0.054). Stratified by lesion number category, the recurrence group had significantly higher proportions of patients with > 20 lesions and 11–20 lesions (*P* = 0.015). Among treatment-related factors, treatment course completion (*P* = 0.040) and treatment response (*P* = 0.002) differed significantly between groups. The recurrence group had a higher proportion of incomplete treatment courses (25.93% vs. 12.77%) and a significantly higher rate of partial clearance (57.41% vs. 30.85%) (**Table 1**).

**Table 1:** Comparison of baseline clinical characteristics.

Variable	Overall	Recurrence group (n = 54)	Non-recurrence group (n = 94)	*t/χ²*	*P*
Age (years)	69.43 ± 7.58	70.96 ± 7.25	68.55 ± 7.66	1.906	0.057
Lesion count	14.03 ± 8.18	15.76 ± 8.57	13.03 ± 7.81	1.923	0.054
Sex				0.704	0.406
Male	81 (54.730%)	32 (59.259%)	49 (52.128%)		
Female	67 (45.270%)	22 (40.741%)	45 (47.872%)		
Skin phototype				0.897	0.346
Light	126 (85.135%)	44 (81.481%)	82 (87.234%)		
Dark	22 (14.865%)	10 (18.519%)	12 (12.766%)		
Immunosuppression				0.926	0.338
No	131 (88.514%)	46 (85.185%)	85 (90.426%)		
Yes	17 (11.486%)	8 (14.815%)	9 (9.574%)		
Prior NMSC history				0.258	0.618
No	121 (81.757%)	43 (79.630%)	78 (82.979%)		
Yes	27 (18.243%)	11 (20.370%)	16 (17.021%)		
Lesion count category				8.399	0.015
5-10	67 (45.270%)	16 (29.630%)	51 (54.255%)		
11-20	53 (35.811%)	25 (46.296%)	28 (29.787%)		
> 20	28 (18.919%)	13 (24.074%)	15 (15.957%)		
Location (sun-exposed)				0.918	0.340
No	45 (30.405%)	19 (35.185%)	26 (27.660%)		
Yes	103 (69.595%)	35 (64.815%)	68 (72.340%)		
Lesion morphology				1.368	0.240
Thin	94 (63.514%)	31 (57.407%)	63 (67.021%)		
Non-thin	54 (36.486%)	23 (42.593%)	31 (32.979%)		
Initial treatment				6.113	0.189
Local laser therapy	54 (36.486%)	21 (38.889%)	33 (35.106%)		
PDT	39 (26.351%)	9 (16.667%)	30 (31.915%)		
Oral retinoids	16 (10.811%)	5 (9.259%)	11 (11.702%)		
Imiquimod	19 (12.838%)	10 (18.519%)	9 (9.574%)		
Combination/Sequential	20 (13.514%)	9 (16.667%)	11 (11.702%)		
Course completed				4.102	0.040
Yes	122 (82.432%)	40 (74.074%)	82 (87.234%)		
No	26 (17.568%)	14 (25.926%)	12 (12.766%)		
Treatment response				10.034	0.002
Complete	88 (59.459%)	23 (42.593%)	65 (69.149%)		
Partial	60 (40.541%)	31 (57.407%)	29 (30.851%)		
Regular sunscreen use				2.783	0.091
No	94 (63.514%)	39 (72.222%)	55 (58.511%)		
Yes	54 (36.486%)	15 (27.778%)	39 (41.489%)		

NMSC, Non-melanoma skin cancer; PDT, Photodynamic therapy.

### 3.2 Incidence and temporal distribution of local recurrence

**Figure 1:**
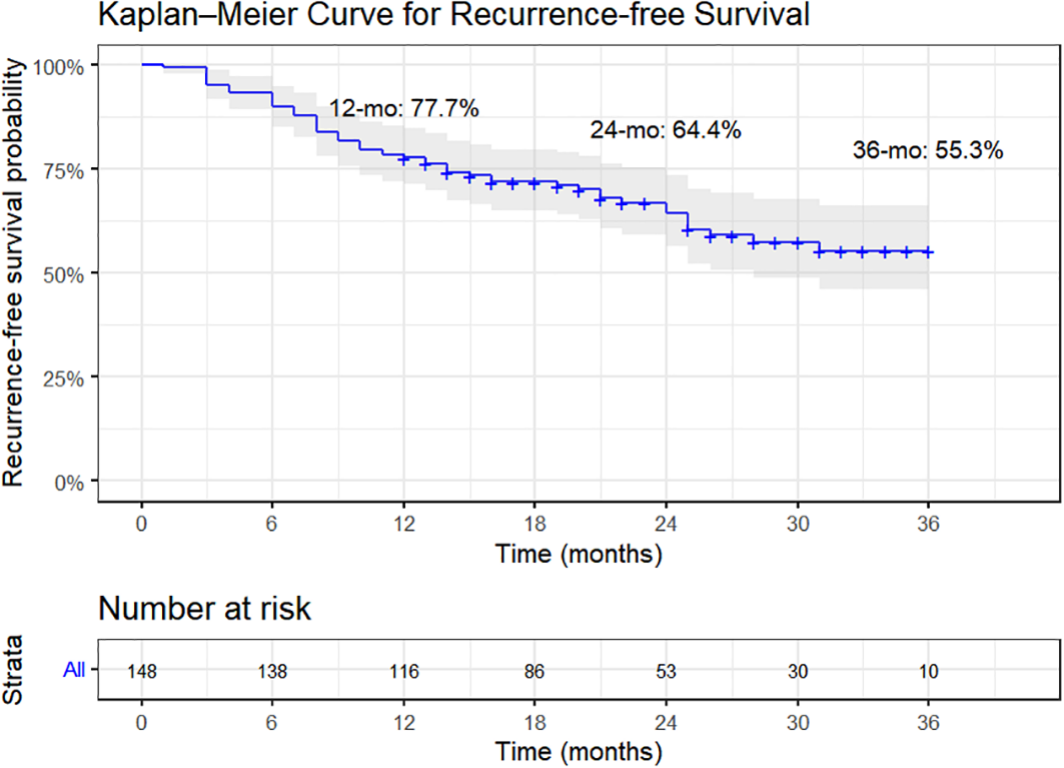
Recurrence-free survival curve for patients with multiple AKs.

Recurrence events were concentrated in the early post-treatment period. The cumulative recurrence rates at 12, 24, and 36 months were 22.3%, 35.6%, and 44.7%, respectively, indicating a gradually increasing risk over time. The median RFS was not reached (as over half of the patients did not experience recurrence during follow-up) (**Table 2**). The Kaplan-Meier curve showed that most recurrences occurred within the first 12 months post-treatment, after which the risk increase plateaued (**Figure 1**).

**Table 2:** Temporal distribution and cumulative recurrence rates.

Time (months)	Number at risk (n)	Events (cumulative, n)	Cumulative recurrence rate (%)	RFS rate (%)
12	116	33	22.3	77.7
24	53	48	35.6	64.4
36	10	54	44.7	55.3

RFS, Recurrence-free survival.

### 3.3 Analysis of risk factors for local recurrence

Using local recurrence as the dependent variable, Cox proportional hazards regression models were employed. Multivariate analysis included host-, lesion-, and treatment-related factors prespecified based on previous literature (9–11) and clinical relevance, along with variables with P< 0.10 in univariate analysis. Lesion count was entered into the Cox regression model as a categorical variable, with patients with 5–10 lesions serving as the reference group and those with 11–20 and >20 lesions modeled as separate categories.

In univariate Cox regression analysis, age, lesion number category (11–20 vs. 5–10), and treatment response were significantly associated with recurrence risk (all P< 0.05) (**Table 3**). Each 1-year increase in age was associated with a 3.6% increase in recurrence risk (HR = 1.036, 95% CI 1.001–1.072, P = 0.045). Patients with 11–20 lesions had a significantly higher risk of recurrence compared with those with 5–10 lesions (HR = 1.765, 95% CI 1.032–3.017, P = 0.038). Incomplete treatment response was also associated with an increased risk of recurrence (HR = 2.378, 95% CI 1.383–4.086, P = 0.002). Regular sunscreen use was not statistically significantly associated with recurrence risk in univariate analysis (HR = 0.636, 95% CI 0.350–1.154, P = 0.136).

**Table 3:** Univariate Cox regression analysis for local recurrence.

Variable	*B*	*SE*	*Wald*	*P*	*HR*	95% CI lower	95% CI upper
Age (per 1 year)	0.035	0.018	4.016	0.045	1.036	1.001	1.072
Immunosuppression (yes vs. no)	0.374	0.383	0.952	0.329	1.454	0.686	3.082
Lesions 11–20 vs. 5-10	0.568	0.274	4.311	0.038	1.765	1.032	3.017
Lesions > 20 vs. 5-10	0.353	0.319	1.226	0.268	1.423	0.762	2.657
Treatment response (partial vs. complete)	0.866	0.276	9.825	0.002	2.378	1.383	4.086
Regular sunscreen (yes vs. no)	-0.453	0.304	2.219	0.136	0.636	0.35	1.154
Location (exposed vs. non-exposed)	-0.175	0.285	0.376	0.540	0.839	0.48	1.469
Morphology (non-thin vs. thin)	0.414	0.276	2.247	0.134	1.513	0.88	2.599
Prior NMSC history (yes vs. no)	0.096	0.338	0.081	0.776	1.101	0.568	2.136

NMSC, Non-melanoma skin cancer; SE, Standard error; HR, Hazard ratio; CI, Confidence interval; B represents the Cox regression coefficient on the log-hazard scale.

In the multivariable Cox regression model, lesion burden remained one of the strongest independent predictors of local recurrence (**Table 4**). Compared with patients with 5–10 lesions, those with 11–20 lesions (HR = 2.390, 95% CI 1.269–4.503, P = 0.007) and those with more than 20 lesions (HR = 2.957, 95% CI 1.367–6.395, P = 0.006) had a significantly increased risk of recurrence. Incomplete treatment response also remained independently associated with recurrence risk after adjustment (HR = 2.431, 95% CI 1.387–4.263, P = 0.002).

**Table 4:** Multivariate Cox regression analysis for local recurrence.

Variable	*B*	*SE*	*Wald*	*P*	*HR*	95% CI lower	95% CI upper
Immunosuppression (yes vs. no)	0.521	0.399	1.706	0.192	1.684	0.77	3.684
Lesions 11–20 vs. 5-10	0.871	0.323	7.274	0.007	2.39	1.269	4.503
Lesions > 20 vs. 5-10	1.084	0.394	7.588	0.006	2.957	1.367	6.395
Treatment response (partial vs. complete)	0.888	0.287	9.617	0.002	2.431	1.387	4.263
Regular sunscreen (yes vs. no)	-0.413	0.308	1.797	0.180	0.661	0.361	1.210

SE, Standard error; HR, Hazard ratio; CI, Confidence interval; B represents the Cox regression coefficient on the log-hazard scale.

Immunosuppression and regular sunscreen use, which were included in the multivariable model as clinically relevant covariates based on prior evidence, were not independently associated with recurrence risk after adjustment (immunosuppression: HR = 1.684, P = 0.192; regular sunscreen use: HR = 0.661, P = 0.180). These findings indicate that, after accounting for lesion burden and treatment response, regular sunscreen use did not demonstrate an independent protective effect against local recurrence in this cohort.

### 3.4 Subgroup and interaction effect analysis

To further explore differences in recurrence risk across clinical subgroups, stratified and interaction effect analyses were conducted. Stratified Cox regression by treatment response status showed that lesion count was positively associated with recurrence risk in both the complete clearance (HR = 1.037, 95% CI 0.996-1.080) and partial clearance (HR = 1.042, 95% CI 0.994-1.093) groups, suggesting increased risk with higher lesion counts, albeit with similar magnitudes of increase between groups.

Subsequently, an interaction term (lesion count × treatment response) was included in the Cox model. The interaction term was not statistically significant (B = 0.005, HR = 1.005, 95% CI 0.944-1.070, *P* = 0.874) (**Table 5**), indicating no significant interaction between lesion number and treatment response regarding their effect on recurrence risk. This suggests that treatment response did not significantly modify the effect of lesion number.

**Table 5:** Cox regression analysis of lesion count, treatment response, and their interaction on local recurrence risk.

Model type	Variable	B	SE	Wald	*P*	HR	95% CI lower	95% CI upper
Interaction model (overall)	Lesion count	0.036	0.021	3.044	0.081	1.037	0.996	1.080
	Response (partial vs. complete)	0.932	0.574	2.643	0.104	2.540	0.825	7.818
	Lesion count × response	0.004	0.032	0.014	0.905	1.004	0.943	1.068
Stratified model (complete)	Lesion count	0.036	0.021	3.071	0.080	1.037	0.996	1.080
Stratified model (partial)	Lesion count	0.041	0.024	2.929	0.087	1.042	0.994	1.093

SE, Standard error; HR, Hazard ratio; CI, Confidence interval; B represents the Cox regression coefficient on the log-hazard scale.

**Figure 2:**
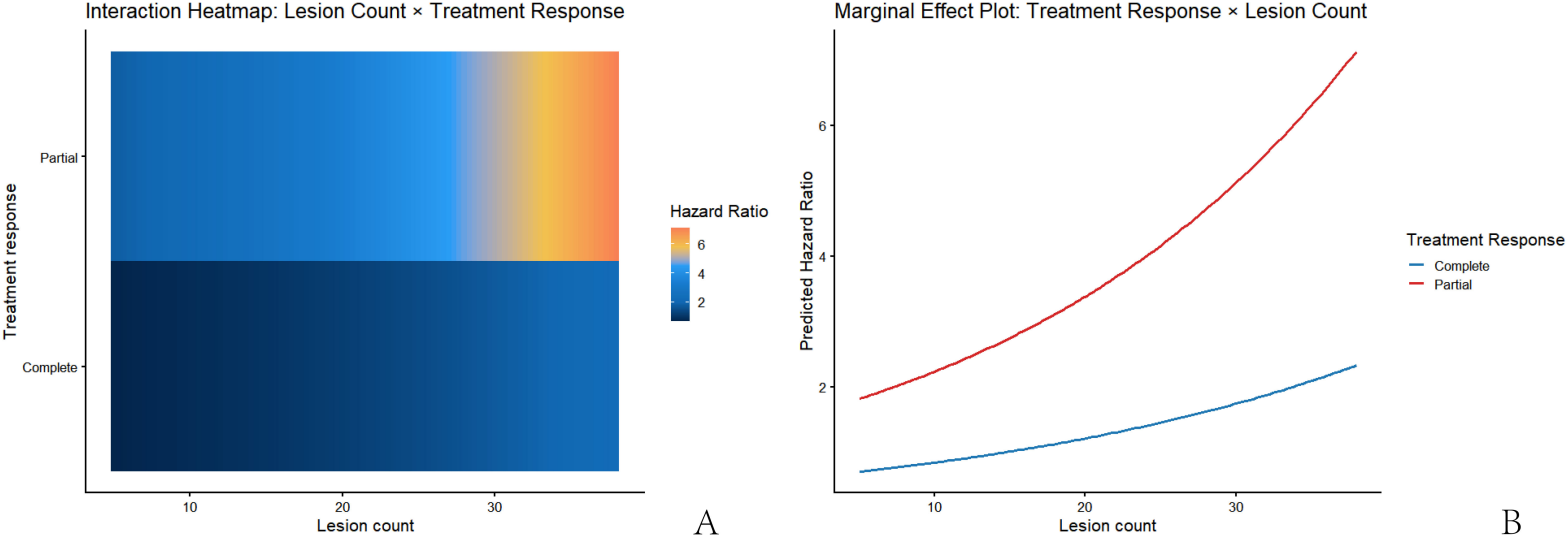
Interaction effect analysis between lesion count and treatment response on local recurrence risk. **(A)** Heat map of the interaction effect; **(B)** Marginal effect plot of predicted recurrence risk.

Despite the non-significant interaction term, heat maps and marginal effect plots were generated to visualize trend variations across different ranges (**Figures 2A, B**). The heat map indicated that predicted recurrence risk gradually increased with lesion number in the partial clearance group, while the increase was relatively milder in the complete clearance group. The marginal effect plot further confirmed that the risk curve for the partial clearance group was consistently higher than that for the complete clearance group, but the slopes were similar, consistent with the non-significant interaction term. These findings collectively indicate that lesion number is a shared risk factor in both groups, but treatment response did not significantly alter the strength of its association with recurrence risk.

### 3.5 Model validation and predictive analysis

**Figure 3:**
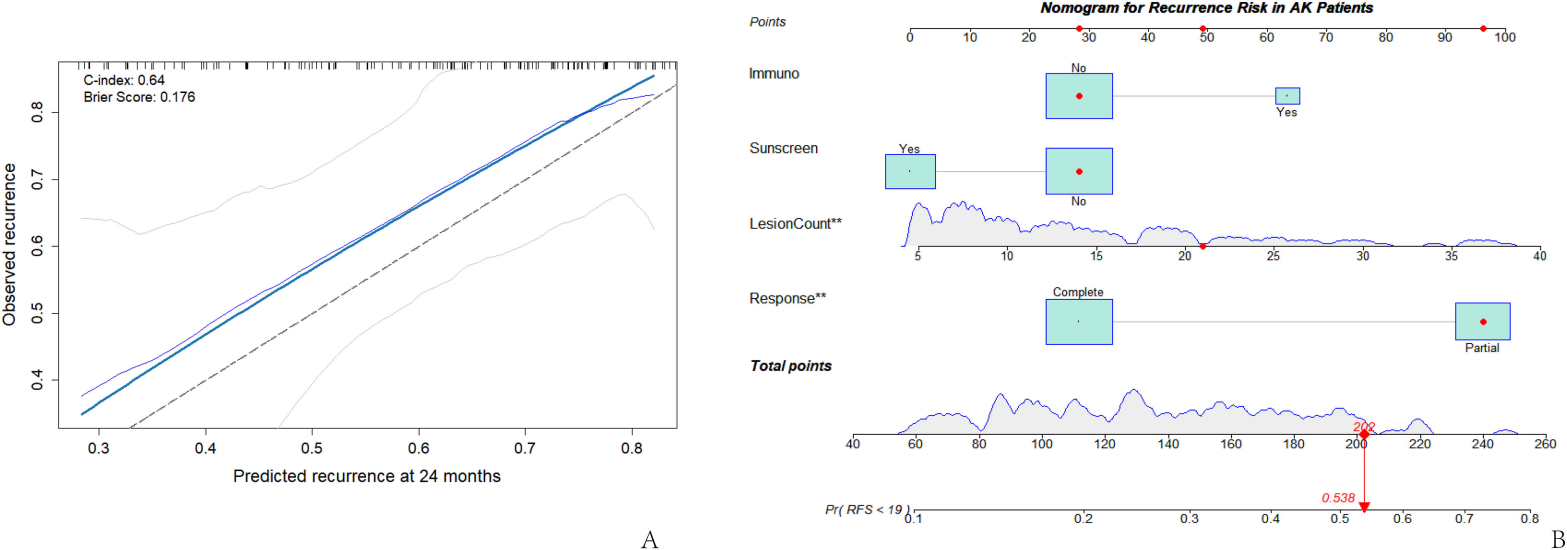
Predictive performance and risk visualization of the multivariate Cox model. **(A)** Calibration curve of the multivariate Cox model; **(B)** Nomogram for predicting local recurrence risk in patients with multiple AKs.

The discriminative ability and calibration of the multivariate Cox model were further validated. The Harrell’s C-index of the model was 0.640, indicating moderate predictive ability for distinguishing between patients with and without local recurrence. The 24-month calibration curve, constructed using bootstrap resampling (B = 200), showed good agreement between predicted and observed recurrence probabilities (**Figure 3A**), indicating good calibration. Furthermore, the model’s Brier score was 0.176, suggesting low overall prediction error and supporting the model’s stability and reliability. Based on the multivariate Cox model, a nomogram for predicting local recurrence risk was constructed (**Figure 3B**). The nomogram integrated key predictors such as immunosuppression status, lesion number category, treatment response, and regular sunscreen use. By summing the points assigned to each variable, the total points corresponded to an individualized probability of recurrence.

## 4 Discussion

AK is a clinical manifestation of field cancerization, characterized by multiple visible and subclinical lesions within a photodamaged field, carrying the potential for recurrence and progression to SCC (12–14). Although various treatments like cryotherapy can effectively clear individual lesions, recurrence is common, and risk varies considerably among patients. Therefore, assessing factors associated with recurrence and developing risk prediction models hold significant clinical value for guiding individualized follow-up and precise treatment.

Based on a real-world cohort of patients with multiple AKs, this study delineated the temporal pattern of local recurrence and identified key influencing factors. The cumulative recurrence rates at 12, 24, and 36 months were 22.3%, 35.6%, and 44.7%, respectively, with most events occurring within 12–24 months post-treatment, consistent with previous reports on the timeline of AK recurrence (15). Relevant European multicenter data also indicate that recurrences typically occur within the first two years post-treatment, often earlier and more concentrated in areas of greater photodamage (16, 17). Our observations further support that AK is a chronic photodamage disease based on field cancerization, where recurrence reflects both the reactivation of residual lesions and the continuous development of new lesions within the compromised skin field. This interpretation is consistent with prospective long-term studies that more comprehensively capture field cancerization, repeated treatment cycles, and cumulative keratinocyte carcinoma burden. Such studies indicate that recurrence over extended follow-up often reflects ongoing field failure rather than true relapse of a single treated lesion (18). Accordingly, the recurrence rates observed in our study likely represent a composite of residual lesion reactivation and early emergence of new lesions within a chronically damaged field.

Importantly, the treatment patterns represented in this cohort (2019–2022) should be interpreted in light of evolving real-world management strategies for actinic keratoses. In recent years, short-course, field-directed therapies with high adherence, such as tirbanibulin 1% ointment, have been increasingly adopted in clinical practice. Large real-world evidence has demonstrated favorable treatment completion rates and sustained effectiveness with tirbanibulin, particularly compared with longer topical regimens that are more susceptible to non-completion in routine care (19). Consequently, predictors of treatment completion and response identified in the present study may differ in magnitude when applied to contemporary cohorts treated predominantly with short-duration, high-adherence field therapies.

Mechanistically, UV radiation (particularly UVB) causes direct DNA damage to epidermal keratinocytes, leading to the accumulation of driver mutations in genes like TP53, NOTCH1, CDKN2A, and FGFR3, forming multiple latent mutant clones. These clones do not progress synchronously but expand gradually, driven by the local microenvironment, inflammatory responses, and declining skin repair capacity, giving rise to clinical and subclinical lesions (20). Previous high-throughput sequencing studies have revealed high similarity in mutational profiles and clonal architecture between AK lesions, photodamaged epidermis, and early cutaneous SCC, suggesting that AK is inherently a chronic, recurrent disease with polyclonal subpopulations (21). This implies that clinical recurrence likely reflects the re-expansion of previously residual or subclinical clones rather than entirely new events. The observed recurrence peak within the first two years in our study aligns with this clonal kinetic model.

In this study, the number of lesions was a core predictor of local recurrence, with higher numbers correlating with increased risk. Previous research indicates that lesion count indirectly reflects the extent of compromised skin and the degree of field cancerization, serving as a quantitative measure of photodamage burden and representing the number and competitive pressure of mutant clones (22). Consequently, even if surface lesions are cleared by initial treatment, deeper or marginal mutant clones may continue to expand, increasing recurrence potential. The marginal effect analysis in our study reinforces this point: the risk-amplifying effect of incomplete treatment response became more pronounced with higher lesion numbers, suggesting that patients with high lesion loads require greater attention to the adequacy of lesion clearance post-treatment.

Incomplete treatment response was another independent high-risk factor for recurrence, with a clear pathological basis. Although modalities like PDT, chemical peels, and cryotherapy effectively target abnormal keratinization, their depth of effect is limited. They may fail to eradicate lesions with hyperkeratosis, associated superficial dermal inflammation, or areas with heterogeneous mutations. The presence of numerous subclinical mutant clones in field-cancerized skin, which prior studies suggest have a higher potential for expansion after superficial therapies (23), explains the significantly higher recurrence risk in patients with incomplete response, consistent with our findings. In addition, lesion- and field-directed pre-conditioning represents a practical but unmeasured determinant of treatment effectiveness in real-world AK management. Randomized evidence has shown that keratolytic pretreatment, such as 10% urea applied before methyl aminolevulinate photodynamic therapy (MAL-PDT), significantly enhances protoporphyrin IX (PpIX) uptake and improves clinical outcomes, particularly in hyperkeratotic AKs (24). The lack of detailed information on pre-conditioning strategies in the present cohort may partly contribute to heterogeneity in treatment response and subsequent recurrence risk. Immunosuppression is a potential risk factor for recurrence but did not reach statistical significance in this cohort. Possible explanations include the low proportion of immunosuppressed patients, limiting statistical power. Furthermore, while sun protection mechanistically reduces cumulative photodamage, its subjective nature and the difficulty in reversing long-term photodamage might explain why regular sunscreen use did not show a significant protective effect in this study.

Regarding model performance, the multivariate Cox model demonstrated moderate discriminative ability (Harrell’s C-index = 0.638), consistent with the multifactorial nature of AK. The calibration curve indicated good predictive accuracy at 24 months, closely matching the observed recurrence rates. This suggests that the model reliably represents the risk structure for recurrence during medium-term follow-up. The nomogram further digitizes the model into a visual scoring system, potentially enabling risk stratification based on lesion number, treatment response, immune status, and sunscreen use, and may assist clinical risk assessment.

In terms of clinical application, the model’s value is threefold: First, it aids in formulating individualized follow-up strategies, suggesting shorter intervals for high-risk patients (e.g., those with > 20 lesions or incomplete response). Second, it can guide treatment selection; for instance, patients with extensive field cancerization might benefit from field-directed therapies with broader coverage and greater depth, such as 5-aminolevulinic acid PDT, imiquimod, or sequential/combination regimens. Third, it can be used for patient education and management, as quantified recurrence risk may improve adherence to sun protection and long-term management.

## 5 Study significance and limitations

This study underscores the central role of lesion burden and treatment response in AK recurrence, providing plausible clinical and biological explanations. It also proposes a predictive model for recurrence risk that may support individualized follow-up and risk assessment in patients with multiple AKs.

Nevertheless, several limitations should be acknowledged. First, this was a single-center study with a relatively limited sample size, which may introduce selection bias and limit generalizability. In addition, the absence of novel biomarkers, such as genetic mutation profiles or advanced skin imaging parameters (e.g., reflectance confocal microscopy), may have constrained the predictive performance of the model. Future studies should focus on external validation and explore the integration of multi-omics data and artificial intelligence–assisted imaging to enhance risk prediction. Second, although multivariable Cox regression was applied, the relatively limited number of recurrence events may increase the risk of model overfitting, particularly in subgroup, interaction, and nomogram analyses. Accordingly, findings from subgroup and interaction analyses should be interpreted as exploratory and hypothesis-generating rather than confirmatory. Finally, several limitations inherent to retrospective real-world cohorts warrant consideration. Local recurrence was operationally defined as the appearance of an AK lesion within a 2 cm radius of the treated area; in the context of field cancerization, this definition may partially capture newly emerging lesions rather than true local relapse, potentially leading to overestimation of recurrence. In addition, treatment-selection bias (confounding by indication) is unavoidable, as treatment choice and completion are influenced by lesion thickness, field severity, anatomical site, seasonality, and patient preference. Although multivariable adjustment was performed, residual confounding cannot be fully excluded.

## 6 Conclusion

While our Cox-based nomogram provides clinically relevant risk stratification for local recurrence in patients with multiple AKs, its generalizability should be interpreted with caution. External validation in contemporary cohorts incorporating evolving treatment paradigms and real-world confounders is warranted.

## Data Availability

The original contributions presented in the study are included in the article/supplementary material. Further inquiries can be directed to the corresponding authors.
